# Atrial fibrillation following cardiac surgery: risk analysis and long-term survival

**DOI:** 10.1186/1749-8090-7-87

**Published:** 2012-09-19

**Authors:** Solveig Helgadottir, Martin I Sigurdsson, Inga L Ingvarsdottir, David O Arnar, Tomas Gudbjartsson

**Affiliations:** 1 Departments of Cardiothoracic Surgery, University of Iceland, Reykjavik, Iceland; 2 Cardiology of Landspitali University Hospital, University of Iceland, Reykjavik, Iceland; 3 Faculty of Medicine, University of Iceland, Reykjavik, Iceland

**Keywords:** Postoperative atrial fibrillation, Coronary artery bypass surgery, Atrial valve replacement, Risk factors, Risk assessment, Prophylaxis, Survival

## Abstract

**Background:**

We studied potential risk factors for postoperative atrial fibrillation (POAF) in a large cohort of patients who underwent open-heart surgery, evaluating short- and long-term outcome, and we developed a risk-assessment model of POAF.

**Methods:**

A retrospective study of 744 patients without prior history of AF who underwent CABG (n = 513), OPCAB (n = 207), and/or AVR (n = 156) at Landspitali Hospital in 2002–2006. Logistic regression analysis was used to study risk factors for POAF, comparing patients with and without POAF.

**Results:**

The rate of POAF was 44%, and was higher following AVR (74%) than after CABG (44%) or OPCAB (35%). In general, patients with POAF were significantly older, were more often female, were less likely to be smokers, had a lower EF, and had a higher EuroSCORE. The use of antiarrythmics was similar in the groups but patients who experienced POAF were less likely to be taking statins. POAF patients also had longer hospital stay, higher rates of complications, and operative mortality (5% vs. 0.7%). In multivariate analysis, AVR (OR 4.4), a preoperative history of cardiac failure (OR 1.8), higher EuroSCORE (OR 1.1), and advanced age (OR 1.1) were independent prognostic factors for POAF. Overall five-year survival was 83% and 93% for patients with and without POAF (p <0.001).

**Conclusion:**

POAF was detected in 44% of patients, which is high compared to other studies. In the future, our assessment score will hopefully be of use in identifying patients at high risk of POAF and lower complications related to POAF.

## Background

Atrial fibrillation (AF) is common following open-heart surgery. While postoperative atrial fibrillation (POAF) can be transient and without consequences, it may lead to serious complications such as increased risk of acute kidney injury (AKI), hemodynamic instability, cardiac failure, stroke, and death [1-3]. As highlighted by the significantly increased cost of treating patients with this arrhythmia and its consequences [4], substantial resources are devoted to detection and treatment of POAF [5,6]. Furthermore, as the rate of POAF increases with age, POAF will present an increasing problem in the large population of elderly patients that undergo cardiac surgery [7,8].

Reported rates of POAF after surgical revascularization and atrial valve replacement (AVR) range widely, from 3% to 90%, but with most of them in the 20–40% range [9,10]. This variation is related to different patient populations, methods and duration of arrhythmia surveillance, and use of medication for prophylactic purposes. POAF is most often detected on the second postoperative day and is frequently self-limiting and short-lived [5]. Up to 80% of patients convert to sinus rhythm (SR) within 24 h, and six weeks after initial diagnosis 98% of patients have converted to SR [11].

Several risk factors for POAF have been reported, such as advanced age, genetic predisposition, chronic obstructive pulmonary disease (COPD), heart failure, valvular surgery, increased perioperative ischemia, and postoperative pneumonia [5,9,12]. Both pharmacological treatment (e.g. beta-blockers and amiodarone) [13,14] and non-pharmacological (e.g. atrial pacing) [15-18] have been used to treat and prevent POAF, but all therapeutic options have variable efficacy and some may adversely affect hemodynamic stability.

Identification of patients at high risk of POAF after cardiac surgery is vital for selection of the patients who might benefit from intensive prophylactic therapy or increased monitoring. Thus, an accurate model predicting the risk of POAF might help to define this challenging group preoperatively. We evaluated potential risk factors for POAF in a large nationwide cohort of patients who underwent myocardial revascularization with or without AVR. In addition, we studied short-term complications, 30-day mortality, and long-term survival.

## Methods

This was a retrospective whole-population study of all patients who underwent off-pump coronary artery bypass grafting (OPCAB), CABG, and AVR for aortic stenosis at Landspitali University Hospital in Iceland, between January 1, 2002 and December 31, 2006. The hospital is the sole institution performing open-heart surgery in Iceland and since 1986 over 5,500 open-heart procedures have been performed.

Patients were identified through two separate registries. First, a computerized diagnosis and operation registry was checked for patients who underwent coronary artery bypass grafting (CABG), OPCAB, and/or AVR with either biological or mechanical prosthesis. Secondly, a centralized cardiac surgery database at our institution was used to identify operated patients, thus confirming a 100% match with the subset identified in the initially mentioned registry.

Altogether, 876 patients underwent the surgeries mentioned above, representing approximately 87% of all patients who underwent cardiac surgery in Iceland during the five-year period. Of these, 207 were OPCAB patients (24%), 507 underwent CABG (58%), 136 (18%) had aortic valve surgery, and 20 (2%) had both aortic valve surgery and coronary revascularization. Altogether, 132 patients were excluded, most often due to a preoperative history of AF (n = 109). None of the patients died intraoperatively. This left 744 patients for further analysis.

Patients with POAF were compared with patients with postoperative normal sinus rhythm (NSR). POAF was defined as AF diagnosed with a rhythm monitor/telemetry and/or ECG, with duration of ≥ 5 minutes and/or initiation of treatment for atrial fibrillation such as amiodarone or cardioversion.

Clinical information was obtained from patient charts and surgical reports, was registered in a standardized data sheet using Excel (Microsoft Corp., Redmond, WA), and was reviewed by three of the authors (S.H., I.L.I., and T.G.). Over 200 variables were registered, including gender, age, cardiovascular risk factors, history of arrhythmia and myocardial infarction. Information on left ventricular ejection fraction (EF) and medication was also collected, including anti-arrhythmic drugs such as beta-blockers, cholesterol-lowering statins, and anticoagulation or antiplatelet drugs. Patients’ symptoms were evaluated according to the New York Heart Association classification and their EuroSCORE (European System for Cardiac Operative Risk Evaluation) calculated [19]. In addition, information on the degree of coronary artery disease (i.e. three-vessel disease, left main stem stenosis), acute vs. elective surgery, cardiopulmonary bypass (CPB), cross-clamp time, and skin-to-skin operative time was registered.

Hospital morbidity was assessed by means of intraoperative and postoperative complications (minor/major) and length of stay. Operative mortality was defined as the number of patients who died within 30 days of surgery. Apart from AF, postoperative complications were categorized as either minor or major. Minor complications included leg wound infection, urinary tract infection, and pneumonia and major complications stroke, mediastinitis, endocarditis, and myocardial infarction (MI) (defined as isolated ST segment changes or a new left bundle branch block on ECG along with an elevation of CK-MB of ≥70 μg/L), AKI as defined by the RIFLE criteria [20] and necessitating renal replacement therapy, reoperation, sternum dehiscence, and acute respiratory distress syndrome (ARDS) or multiple organ failure (MOF). We also recorded bleeding (defined as the 24-hour postoperative chest tube output) and number of transfusions of packed red cells (PRC).

Patients were assigned a date and a cause of death or were identified as still living on September 1, 2010, using data from the Icelandic National Population Registry. Overall survival was calculated using the Kaplan-Meier method. Mean follow-up time was 60 months (range: 0–97 months) and none of the patients were lost to follow-up.

### Statistical methods

Continuous variables were compared between the groups with t-test or Mann–Whitney test, depending on whether the data were normally distributed, and categorical variables were compared using either Fisher’s exact test or Chi-square test. Survival was plotted on a Kaplan-Meier curve and the groups were compared with a log-rank test. A multivariate logistic regression model of independent risk factors for POAF was pursued using variables from the univariate analysis with p-values less than 0.1 as predictor variables, and reducing the model using a semi-automated stepwise backwards method until the best model was found. The predictive capabilities of the finalized model were assessed by calculating the area under the receiver operating characteristic (ROC) curve and a Cox proportional hazard model assessed the contribution of variables to long-term survival. All variables in the finalized model met requirements of proportionality. Both odds ratios (ORs) in the logistic models and hazard ratios (HRs) in the Cox model are reported, along with 95% confidence intervals. The level of statistical significance was set at p <0.05. All statistical analysis was performed with R software version 2.12.1 (The R Foundation, Austria) using the survival, MASS, and pROC packages.

The study was approved by the Icelandic National Bioethics Committee and the Icelandic Data Protection Commission. As individual patients were not identified, obtaining individual consent for the study was waived.

## Results

The overall incidence of POAF was 44%, and was significantly higher in patients who underwent AVR or combined AVR and CABG compared to those who underwent myocardial revascularization alone (74%, 81%, and 37%, respectively; p < 0.001). Incidence of POAF was significantly lower in patients who underwent OPCAB than in those who underwent conventional CABG (35% vs. 46%, p = 0.01).

Patient demographics are compared in Table 1. Patients who experienced POAF were more often female and on average 6 years older than patients with NSR. They were more likely to have chronic heart failure but less likely to have dyslipidemia or a history of smoking. There was no significant difference between groups regarding diabetes or hypertension. Significantly fewer coronary vessels were affected in the POAF group, but their EF was lower and their EuroSCORE was higher. There was no significant difference in preoperative use of beta or calcium channel blockers between groups but statin use was less common in the POAF group (63% vs. 76%, p < 0.001).

**Table 1:** Comparison of patient demographics for patients with POAF and NSR following CABG/OPCAB ± AVR in Iceland between 2002 and 2006

	**POAF (n = 326)**	**NSR (n = 418)**	**p-value**
Male (%)	244 (74)	349 (83)	0.005
Age in years, mean ± SD	70 ± 9.3	64 ± 8.7	< 0.001
Diabetes (%)	62 (19)	60 (14)	0.11
Hypertension (%)	213 (65)	248 (59)	0.11
Dyslipidemia (%)	161 (49)	263 (63)	< 0.001
Smoking history (%)	58 (18)	111 (35)	0.005
Chronic heart failure (%)	66 (20)	35 (8)	< 0.001
Mean number of affected vessels	2.4	2.7	< 0.001
Mean left ventricular ejection fraction (LVEF)	51	55	< 0.001
Mean EuroSCORE	6.0	4.0	< 0.001
β blocker use preoperatively (%)	201 (62)	291 (70)	0.074
Ca^2+^ channel blocker use preoperatively (%)	67 (21)	80 (19)	0.58
Statin use preoperatively (%)	201 (63)	316 (76)	< 0.001

Operative factors are compared in Table 2. In CABG patients, the average cardiopulmonary bypass (CPB) time was 15 min longer and aortic cross-clamp time was on average 9 min longer in the POAF group. There was a slight increase in postoperative bleeding, and POAF patients also received more units of PRC.

**Table 2:** Comparison of operation-related factors in patients with POAF and NSR following CABG/OPCAB ± AVR in Iceland between 2002 and 2006

	**POAF (n = 326)**	** NSR (n = 418)**	**p-value**
CABG/OPCAB (%)	233 (37)	391 (63)	< 0.001
AVR (%)	42 (73)	15 (27)	< 0.001
AVR + CABG (%)	51 (81)	12 (19)	< 0.001
OPCAB (%)	270 (46)	313 (54)	0.01
Skin-to-skin time in min, mean (range)	205 (90–640)	200 (100–555)	< 0.001
Time on CPB in min, mean (range)	95 (38–366)	80 (28–265)	< 0.001
Aortic X-clamp time in min, mean (range)	49 (19–209)	40 (13–204)	< 0.001
Chest tube bleeding first 24 h in mL, mean (range)	850 (120–4,980)	773 (120–31,820)	< 0.001
Transfusion of PRC in units, median (range)	2 (0–44)	1 (0–88)	< 0.001

CABG – coronary artery bypass grafting, OPCAB – off-pump coronary artery bypass grafting, AVR – aortic valve replacement, CPB – cardiopulmonary bypass, PRC – packed red cells.

Table 3 lists postoperative complications. A higher ratio of minor infections (pneumonia, superficial wound infections, and urinary tract infections) was seen in patients with POAF and rates of major infections (endocarditis and mediastinitis) were more than fourfold higher (0.9% vs. 0.2%, p = 0.03). Rates of postoperative myocardial infarction, stroke, and renal failure requiring dialysis were not significantly higher in the POAF group but pleural effusion necessitating chest tube drainage was more common in the POAF group. This was also the case for sternum dehiscence (5% vs. 0.7%, p = 0.001) and multiple organ failure or ARDS (8% vs. 0.2%, p <0.001), but reoperation for bleeding was similar (4% vs. 5%, p = 0.9).

**Table 3:** Comparison of minor and major complications, hospital and ICU admission, and operative mortality in patients with either POAF or NSR following CABG/OPCAB ± AVR in Iceland between 2002 and 2006

	**POAF (n = 326)**	**NSR (n = 418)**	**p-value**
Any major complication	59 (25)	70 (18)	0.03
Deep wound infection/mediastinitis/endocarditis (%)	3 (0.9)	1 (0.2)	0.03
Perioperative myocardial infarction (%)	48 (15)	53 (13)	0.34
Stroke (%)	7 (2.1)	6 (1.4)	0.23
Acute kidney injury requiring dialysis (%)	7 (2)	4 (0.9)	0.11
Sternum dehiscence (%)	15 (5)	3 (0.7)	0.24
Multiple organ failure/ARDS (%)	25 (8)	1 (0.2)	< 0.001
Reoperation (%)	14 (4)	20 (5)	0.88
Pneumonia (%)	34 (10)	16 (4)	< 0.001
Superficial wound infection (%)	31 (10)	36 (9)	0.66
Urinary tract infection (%)	26 (8)	11 (3)	0.002
Total hospital stay in days, mean (range)	15 (1–110)	10 (1–47)	< 0.001
Stay in ICU in days, mean (range)	3 (1–13)	1.4 (1–41)	< 0.001
Operative mortality, <30 days (%)	15 (5)	3 (0.7)	0.001

Median length of hospital stay was almost five days longer for patients with POAF, and length of stay in the intensive care unit was more than double that for patients in the NSR group. Operative mortality within 30 days was about seven times higher in patients diagnosed with POAF (5% vs. 0.7%, p = 0.001).

In the finalized multivariate logistic model, the strongest independent risk factor for POAF was AVR surgery (OR 4.36), followed by a history of chronic heart failure (OR 1.81), EuroSCORE (OR 1.1), and advanced age (OR 1.05) (Table 4). The area under the ROC curve for the finalized model was 0.74. Addition of a transformed value of the age (square root, second power, or the natural logarithm) did not change the significance of EuroSCORE (data not shown).

**Table 4:** A finalized logistic regression model of independent risk factors for development of POAF following CABG/OPCAB ± AVR in Iceland between 2002 and 2006

	**Odds ratio**	**95% CI**	**p-value**
Aortic valve replacement	4.36	2.68–7.07	< 0.001
Chronic heart failure	1.81	1.10–2.99	< 0.001
EuroSCORE (per point)	1.10	1.03–1.17	< 0.001
Age (per year)	1.05	1.03–1.07	< 0.001

**Figure 1:**
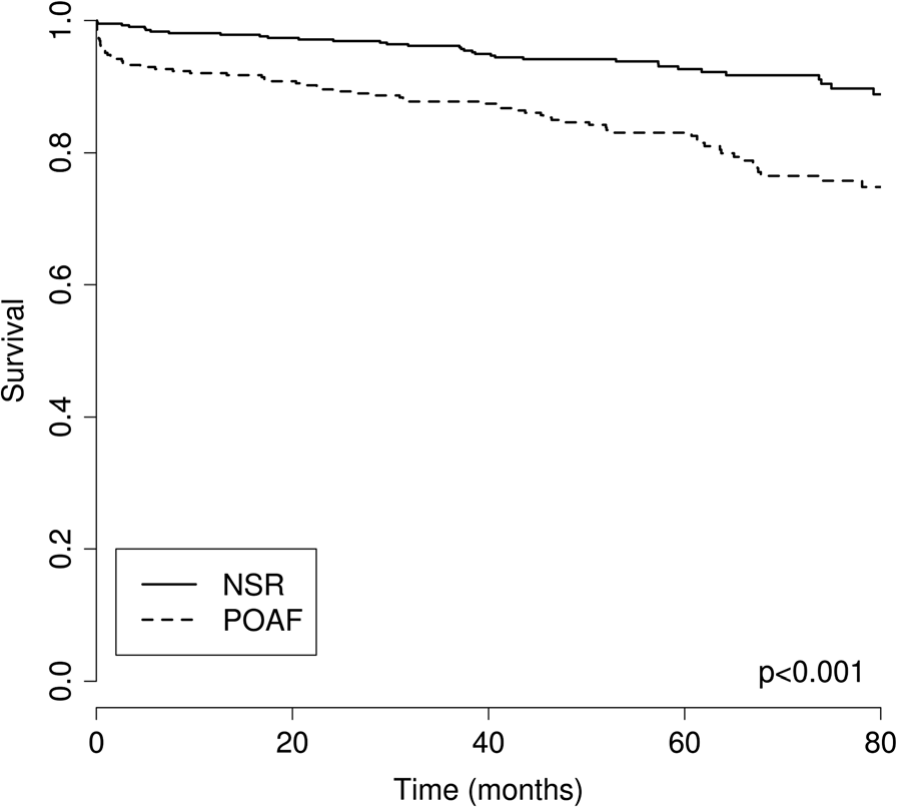
**Probability table of POAF.** A risk score to evaluate the probability of postoperative fibrillation following CABG/OPCAB or AVR + CABG for patients without any prior history of atrial fibrillation, based on age and standardized EuroSCORE. Probability is reported in percentages.

Using the independent risk factors for POAF, we created a risk table that can be used to predict the probability of POAF for patients without prior AF, using preoperative information on operation type, patient age, and standard EuroSCORE (Figure 1). For example, the risk score predicts that a 50-year-old individual undergoing OPCAB operation with a EuroSCORE of 2 has a 17% risk of POAF, while a 75-year-old individual undergoing AVR with a EuroSCORE of 6 has an 82% likelihood of POAF.

**Figure 2:**
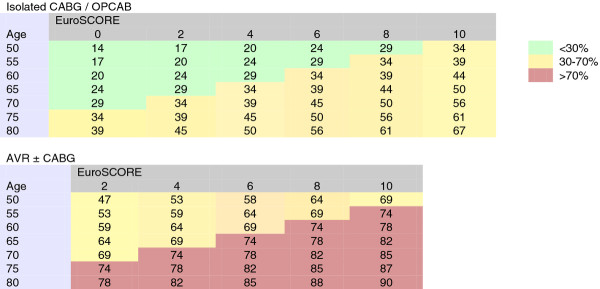
**Survival of patients with POAF and NSR.** A Kaplan-Meyer survival curve of patients with POAF and NSR following CABG/OPCAB, AVR, or CABG/OPCAB + AVR surgery in Iceland between 2002 and 2006.

The long-term survival of POAF patients differed significantly from that of NSR patients (p <0.001, log-rank test) (Figure 2), one- and five-year survival being 92% (95% CI: 89–95%) and 83% (95% CI: 79–87%) for patients with POAF as compared to 98% (95% CI: 97–99%) and 93% (95% CI: 90–95%) in patients with NSR, respectively. The presence of POAF was associated with increased hazard of mortality (HR 2.63, 95% CI: 1.76–3.93, p <0.001), but the HR was reduced in the model (HR 1.64) when corrected for AVR, acute operation, age, and EuroSCORE (Table 4).

## Discussion

Following cardiac surgery in Iceland POAF was found to be common, or 44%. This is a higher rate than found in most other retrospective studies, where it has usually ranged between 20% and 30% [10,21,22]. The reasons for this are not entirely clear. One possible explanation might be the extensive degree of electrocardiographic monitoring that is performed at our institution relative to that done in other studies, i.e. six days of constant telemetry in all patients. As Aranki *et al*. have shown, 70% of POAF occurrence is clustered around the first four postoperative days, 94% being diagnosed in the first 6 days and only 6% more than a week after surgery [5]. Another possible explanation is the lack of systematic use of prophylactic treatment for AF before open-heart surgery at our hospital. Amiodarone therapy was rarely used, and only 20% and 65% of the study patients, respectively, were taking calcium channel blockers and beta-blockers in the immediate postoperative phase. Furthermore, a recent prospective study on the use of omega-3 fatty acids for prevention of POAF in Iceland has led to a couple of interesting observations in this regard. Icelanders have a relatively high baseline levels of omega-3 fatty acids in plasma phospholipids and this particular study demonstrated that higher levels of these fatty acids may actually increase the risk of POAF, contrary to what was previously believed [23].

POAF was more often seen in females and in older patients, especially those with a history of chronic heart failure [5,24]. Furthermore, the higher EuroSCORE and lower EF in the POAF group probably reflect comorbidities that further facilitate the development of POAF. As described in previous studies, the POAF rate was higher in patients who underwent AVR or AVR in combination with surgical revascularization. The nature of AVR surgery may cause structural conduction disturbances and more extensive tissue injury, which might increase the risk of arrhythmias [10].

As in the meta-analysis of Møller *et al*. our univariate analysis showed that patients who underwent OPCAB were less likely to develop POAF than were CABG patients [25]. This may be attributed to the systemic inflammatory effect caused by the CPB, which has in turn been shown to have a pro-fibrillatory effect [24,26].

After surgery, POAF patients received significantly more transfusions of PRC, even though bleeding was only slightly increased. A possible explanation for this is that patients with POAF were believed to be more sensitive to anaemia and loss of cardiac output following surgery. It should, however, be emphasized that timeline and causality were not clear in the study; therefore, it might also be deduced that blood transfusions were pro-fibrillatory in our patients, as shown in previous studies [27].

In the univariate analysis, patients taking statins were less likely to develop POAF. The potential benefit of statin use preoperatively has been extensively researched in patients undergoing cardiac surgery. The effects of statins are still not clear, but randomized trials have shown that they reduce the incidence of POAF following elective cardiac surgery [28].

A number of previous studies have shown an advantage of prophylactic therapy for POAF [29], but owing to their possible negative inotropic effects and a lack of consensus on clinical guidelines, their use has not been universal. Furthermore, patients taking anti-arrhythmic medications before surgery often go days without these drugs postoperatively, due to their potentially negative affects on hemodynamic stability in the early postoperative period, which might further compound the risk of arrhythmias during this period.

The main factors associated with an increased risk of POAF were age, the complexity of the procedure, a history of heart failure, and low EF in addition to a higher EuroSCORE—all factors that have been described in previous studies. This information was used to construct a likelihood table for the development POAF, based on age, operation type, and EuroSCORE (Figure 1). One of the main strengths of our model is the complete dataset that was available for its construction as well as its simplicity, making it a feasible tool for use in clinical practice. The main drawback was the observational design of our study and the low number of study patients, even though incidence was higher than in most previous studies, possibly due to higher pick-up rates. Using the entire population to create the risk model was considered necessary to obtain sufficient power. There is a possibility of overfitting—that the model describes the risk in the population studied adequately but fails to predict the risk in other patient cohorts. The model would be improved by testing out its prediction capabilities with an Icelandic validation cohort, ideally in a prospective manner. We believe that a risk score would help to identify patients who might be candidates for prophylactic therapy or close electrocardiographic monitoring.

Patients with POAF were more likely to be diagnosed with minor complications and any of the major complications, although not all of the individual major complications were significantly more frequent (i.e. stroke, MI, AKI requiring dialysis, and sternum dehiscence). POAF patients also had longer total length of hospital stay, and their stay in ICU was more than doubled. This agrees with the results of previous studies [30].

Postoperative (< 30-day) mortality was 5% in patients who were diagnosed with POAF, as compared to 0.7% in patients with NSR. The difference in survival is also reflected in long-term survival, 5-year survival being 83% for POAF patients and 93% for NSR patients.

This is the first nationwide study of the incidence of POAF following coronary artery and/or AVR surgery. Other strengths of the study were that the patients were found using two separate registries and the phenotype of POAF was well defined, with comprehensive arrhythmia surveillance of longer duration than in most other studies. All patients were operated on and treated for POAF at a single centre, and they were therefore less likely to be affected by bias due to tertiary referral. Furthermore, none of the patients were lost to follow-up. The weakness of the study was its non-randomized nature and the inability to infer causality.

## Conclusion

In summary, this study shows a high incidence of POAF following cardiac surgery. This may in part be explained by a high pick-up rate, but other factors could play a role. In light of the increased short- and long-term risks of patients who develop POAF, it is of particular importance to consider ways to reduce the burden of this arrhythmia. Hopefully, a careful preoperative risk profiling of patients who are particularly likely to experience POAF can aid in selecting patients who are eligible for prophylactic treatment and increased monitoring. To that end, we constructed a thorough risk model that, given further research, might prove suitable in determination of the feasibility of prophylactic therapy.

## Competing interest

The authors declare that they have no competing interests.

## Authors’ contributions

SH, MIS and TG participated in the design of the study. SH, ILI and TG collected data. SH and MIS conducted statistical analysis. MIS, ILI, DOA and TG reviewed literature and drafted the manuscript. SH reviewed literature and wrote the article. All authors read and approved the final manuscript.
